# Estimated assessment of cumulative dietary exposure to organophosphorus residues from tea infusion in China

**DOI:** 10.1186/s12199-018-0696-1

**Published:** 2018-02-14

**Authors:** Pei Cao, Dajin Yang, Jianghui Zhu, Zhaoping Liu, Dingguo Jiang, Haibin Xu

**Affiliations:** 1 National Institute for Nutrition and Health, Chinese Center for Disease Control and Prevention, No 27, Nanwei Road, Xicheng District, Beijing, 100050 China; 2 Risk Assessment of Division One, China National Center for Food Safety Risk Assessment, No 37, Building 2, Guangqu Road, Chaoyang District, Beijing, 100021 China

**Keywords:** Organophosphorus residues, Cumulative dietary exposure, Risk assessment, Tea infusion

## Abstract

**Background:**

China has the world’s largest tea plantation area in the world. To sustain high yields of the tea, multiple pesticides are used on tea crops to control pests. Organophosphorus (OP) pesticides are among the most widely used types of agricultural pesticides in China. As tea is a significant potential source of exposure to pesticide residues, the public concern has increased in relation to pesticide residues found in tea in China. The aim of the study was to estimate cumulative dietary exposure to OP residues from tea infusion for Chinese tea consumers to determine whether exposure to OP residues from tea infusion is a cause of health concern for tea consumers in China.

**Methods:**

OP residue data were obtained from the China National Monitoring Program on Food Safety (2013–2014), encompassing 1687 tea samples from 12 provinces. Tea consumption data were obtained from the China National Nutrient and Health Survey (2002), comprising 506 tea consumers aged 15–82 years. The transfer rates of residues from tea leaves into tea infusions were obtained from the literature. The relative potency factor (RPF) approach was used to estimate acute cumulative exposure to 20 OP residues from tea infusion using methamidophos as the index compound. Dietary exposure was calculated in a probabilistic way.

**Results:**

For tea consumers, the mean and the 99.9th percentile (P99.9) of cumulative dietary exposure to OP residues from tea infusion equalled 0.08 and 1.08 μg/kg bw/d. When compared with the acute reference dose (ARfD), 10 μg/kg bw/d for methamidophos, this accounts for 0.8 and 10.8% of the ARfD.

**Conclusions:**

Even when considering OP residues from vegetables, fruits and other foods, there are no health concerns based on acute dietary exposure to OP residues from tea infusion. However, it is necessary to strengthen the management of the OP pesticides used on tea in China to reduce the risk of chronic dietary exposure to OPs from tea infusion.

**Electronic supplementary material:**

The online version of this article (10.1186/s12199-018-0696-1) contains supplementary material, which is available to authorized users.

## Background

For almost 4000 years, tea has been prepared by processing the leaves of the *Camellia sinensis* plant for consumption in the form of an infusion [1, 2]. China has one of the world’s longest histories of tea production. Depending on how the leaves are processed, three main types of tea can be produced: non-fermented tea, e.g. green tea; semi-fermented tea, e.g. oolong tea; and fermented tea, e.g. black tea [3]. Tea leaves have been found to contain compounds beneficial to human health, such as polyphenols [4, 5]. However, tea leaves may also contain hazardous compounds, such as pesticide residues and mycotoxins [6]. China has the world’s largest tea plantation area and is the second largest tea exporter in the world [7–9]; it exported tea to more than 140 countries from 2005 to 2009 [8, 10]. More and more people around the world are consuming tea because of its beneficial health effects. Clearly, tea is one of the most popular beverages in the world, especially in Asia. Accordingly, the production of tea in China in 2014 totalled 209.2 million tons, an increase of 10.33% over 2013; this accounted for 41.6% of total global production [11]. To sustain such high yields and to ensure the quality of the tea, multiple pesticides are used on tea crops to control pests [12]. Organophosphorus pesticides (OPs) are among the most widely used types of agricultural pesticides in China, owing to their broad-spectrum insecticidal activity, high effectiveness and low cost [13].Some studies have found that a few high toxicity OPs are frequently detected on tea crops in China, although approximately 70% of OPs have been banned due to their high toxicity [14–17].Some old pesticides are still used illegally on tea crops, and tea has a shorter interval between pesticide treatment and harvest compared with other crops [18]. Therefore, tea is a significant potential source of exposure to pesticide residues, particularly among extreme tea consumers (those who consume very high amounts of tea).Thus, public concern has increased in relation to pesticide residues found in tea in China [19–22]. To address this concern, it has become necessary to monitor pesticide residues in tea and to estimate the potential intake of these pesticides.

Conventional approaches to assessing dietary exposure to contaminants and additives in food are deterministic, using point estimates for consumption and contamination [23]. Point estimates are widely used in dietary exposure assessments of pesticide residues in food, as they are simple and accessible [24]. However, they have the obvious limitations of being unrealistic and less informative [25, 26]. The probabilistic approach, in contrast, takes into consideration the uncertainty and variability of exposure and also provides a distribution outcome [27]. It is considered a useful technique for performing acute dietary exposure estimates of pesticide residues [27]. Thus, in some countries and in international organisations, the national registration process for contaminants and additives also includes the probabilistic approach [28].

People are exposed to more than one pesticide through the consumption of tea because tea may contain more than one type of residue [15–17, 29, 30]. Previous dietary exposure assessments of pesticide residues in tea have usually been performed separately for individual pesticides [19, 21, 22]. If these chemicals have the same mode of toxicological action, the traditional method of separately assessing dietary exposure to pesticides may underestimate the health risk [31]. To address dietary exposure to such chemicals, individual exposures should be addressed together. Cumulative exposure assessment is applicable and suitable for assessing the cumulative toxicological effect, which is assumed to be equal to the sum of chemicals’ individual effects if there are no synergistic or antagonistic effects [32, 33]. To calculate the cumulative dietary exposure to OPs, the relative potency factor (RPF) approach was applied in this assessment.

Cumulative dietary exposure assessment to OP residues has been performed in some countries, such as the USA, the Netherlands and Brazil [26, 31, 34]. Meanwhile, Sun J.F. et al. performed a cumulative risk assessment of dietary exposure to OP residues in China, but the food items in this assessment mainly involved vegetables, fruits and staple food; just two kinds of tea was included [35]. There is no report estimating cumulative exposure to OPs for tea consumers on a nationwide scale in China. The purpose of the present study was to estimate cumulative dietary exposure to OP residues from tea infusion for Chinese tea consumers—using a probabilistic approach and the RPF approach—to determine whether exposure to OP residues from tea infusion is a cause of health concern for tea consumers in China.

## Methods

### Pesticide residue data analysis in tea

Pesticide residue data were obtained from the China National Monitoring Program on Food Safety during 2013–2014. A total of 1687 samples from three types of tea were analysed for 25 OP residues. In this monitoring program, the tea samples were collected from supermarkets and local markets in 12 provinces, including Anhui, Fujian, Sichuan, Hunan, Jiangsu, Guangxi, Hubei, Zhejiang, Yunnan, Xinjiang, Beijing and Guangdong in China. Sample analysis was performed by the Center for Disease Prevention and Control in each province or district of China, according to the national standards (GB/T 5009.20-2003) for the determination of OP residues in tea. The analytical method for OPs used gas chromatography (GC)-flame photometric detection (FPD) established by the National Health and Family Planning Commission of the People’s Republic of China. The limit of detection (LOD) ranged from 0.000011 to 0.22 mg kg^−1^. The laboratories were certified for quality control in the detection procedure by China National Center for Food Safety Risk Assessment to ensure the accuracy and comparability of the monitoring data from different laboratories.

### Tea consumption data

The tea consumption data were obtained from the China National Nutrient and Health Survey conducted in 2002 [36]. This survey used multistage, random cluster sampling, which was conducted in 132 sampling locations in 31 provinces in China, using three consecutive, 24-h dietary recall face-to-face interviews, including two weekdays and one weekend, holidays excluded. A total of 506 tea consumers aged 15–82 years (of which 280 were male, 226 were female) recorded their tea consumption volume and types of tea consumed over three consecutive days. In addition, in this survey, the age of the tea consumer was almost always above 15 years old, as children generally do not drink tea. Characteristics of each respondent, including age, sex, weight and address, were obtained through questionnaires.

### Relative potency factors

The RPF approach used the sum of concentrations of various pesticides in the each class after adjustment by RFP. The concentrations of all compounds in a food are expressed as equivalents of one ‘index compound’ (IC) and summed [37].RPFs were defined as the ratio between the critical effect dose (CED) of the IC and the same of the remaining compounds in this group [26]. The CEDs were also selected from international authoritative institutions, e.g. the US Environmental Protection Agency (EPA) and the Joint Meeting of Pesticide Residues (JMPR). In this study, the critical effect was that of AChE inhibition in the female rat brain after 21 days of exposure; this information was provided by the US EPA. As with most OP pesticides, females appeared to be more sensitive than males [34, 38]. The selection of the IC in a cumulative assessment requires many studies and many toxicological data [38, 39]. Methamidophos was used as the IC, as it had high-quality data for toxicological action for AChE inhibition; methamidophos has also been detected in tea samples in China [26, 38]. Additional file 1: Table S1 shows that the RPFs were calculated based on the benchmark dose (BMD) at a 10% AChE reduction in the female rat brain, using methamidophos as the IC.

In addition, Bosgra et al. used the EPA data on AChE inhibition in the female rat brain after 21 days of exposure to fit the dose-response curves [37]. Additional file 1: Table S2 shows that the RPFs were calculated based on CEDs corresponding to 20% AChE inhibition by OPs, derived from the fitted curves using methamidophos as the IC. To analyse whether RPFs derived from different critical effect doses will affect the results of cumulative exposure assessment, both of these CEDs will be used in the calculations.

### Transfer rate of pesticide residues to tea infusion

As tea is consumed indirectly, tea infusion is the main route of human exposure to pesticide residues in tea. Several studies have investigated the transfer rates (TRs) of pesticides during tea brewing [21, 40–44]. The TRs of OP residues from tea leaves into tea infusions were selected from the open scientific literatures, and the details of the TRs of OP residues into tea infusions are shown in Additional file 1: Table S3.

The pesticide residues that were leached into the tea infusion mostly depended on the water solubility and the octanol-water (*K*_ow_) partition coefficient [45–47]. Wan et al. found that, at low water solubility (< 10 mg/kg), the TR was 1–4% and was not sensitive to variations in water solubility. When the water solubility was within the range of 10–150 mg/kg, the TR was sensitive to water solubility. When the water solubility> 179 mg/kg, the TR > 90% and no longer sensitive to changes in water solubility [46]. Additional file 1: Table S4 shows the water solubility and log (*K*_ow_) of OP residues detected in tea samples. According to water solubility and log (*K*_ow_) of OP residues, the OP residues found in tea samples are divided into three categories: soluble pesticides (water solubility ≥ 200 mg/L), moderately soluble pesticides (water solubility 10–200 mg/L) and low soluble pesticides (water solubility< 10 mg/L). Based on the TRs of three types of OP residues obtained from the open scientific literatures, we calculated the median TRs of each type of OP residue to represent the TR of this category. The median TRs of soluble OPs, moderately soluble OPs and low soluble OPs were 87.4, 22.1 and 2.8%, respectively.

### Modelling of cumulative dietary exposure

Acute dietary exposure of Chinese tea consumers to OP residues from tea infusion was calculated in a probabilistic way using a @Risk software (version 6.0, Palisade). The probabilistic modelling software used in this study was implemented by Monte Carlo simulations, which simulated daily consumptions by sampling from the tea consumption distribution and combined these with a random sample from the concentration (the IC equipotent concentrations of OPs adjusted by the corresponding TR in tea infusion) distribution. The random sampling from the concentration distribution was according to the rate of the sample with detectable and rate of the sample with undetectable, respectively. Each tea consumer’s daily cumulative dietary exposure to OP residues from tea infusion (μg/kg bw/d) was calculated by tea consumption combined with the IC equipotent concentrations of OPs in tea infusion on a single day, adjusted by the measured individual body weight of each tea consumer.

The @Risk software was used to fit the concentration data, tea consumption data and body weight data to obtain the appropriate distribution: the *lognorm* distribution for the IC equipotent concentrations of tea samples above LOD. Considering the uncertainty and variability in the concentrations of undetected samples, the concentrations of undetected tea samples were assumed to have a distribution ranging from 0 to the maximum LOD for OPs (0.22 mg/kg), also expressed as the IC equivalents. A *uniform* distribution was assumed for the IC equipotent concentrations of LOD in the case of undetectable samples. The *InvGauss* distribution was applied to tea consumption data. The *normal* distribution was used for tea consumers’ body weights. The details of the descriptions of variables and models for this dietary exposure assessment are shown in Table 1. The number of Monte Carlo iteration was 100,00 and simulation was 200. The exposures were specified at percentiles P50, P90, P95, P97.5, P99 and P99.9 and mean from the intake distribution, and compared with the ARfD of methamidophos, 10 μg/kg bw/d established in 2002 by JMPR [48].

**Table 1:** Description and distribution of variables and models for dietary exposure assessment of OP residues in tea infusion

Compound	Variables	Definition	Assumption/distribution/formula	Source
OPs	*Conc*_*Qp*_	Concentration in methamidophos equivalents of OPs in tea infusion (detected value)	*Lognorm* (0.052, 4.636)^a^	Monitoring data
			*Lognorm* (0.003, 0.041)^b^	
			*Lognorm* (0.003, 0.151)^c^	
	*Conc*_*Qn*_	Concentration in methamidophos equivalents of OPs in tea infusion (undetected value)	*Uniform* (0, 0.105)^a^	Monitoring data
			*Uniform* (0, 0.044)^b^	
			*Uniform* (0, 0.105)^c^	
	*Conc* ^*^_Qp_	Concentrationin in methamidophos equivalents of OPs in tea infusion (detected value)	*Lognorm* (0.038, 2.987)^a^	Monitoring data
			*Lognorm* (0.002, 0.019)^b^	
			*Lognorm* (0.001, 0.019)^c^	
	*Conc* ^*^_Qn_	Concentration in methamidophos equivalents of OPs in tea infusion (undetected value)	*Uniform* (0, 0.447)^a^	Monitoring data
			*Uniform* (0, 0.235)^b^	
			*Uniform* (0, 0.447)^c^	
	*Pp*	Rate of sample with detectable	10.11%^a^	Calculated
			13.53%^b^	
			6.20%^c^	
	*Pn*	Rate of sample with undetectable	89.89%^a^	Calculated
			86.47%^b^	
			93.80%^c^	
	*Conc*_*QC*_	Concentration in methamidophos equivalents of OPs in tea infusion	Discrete (*ConcQp*: *ConcQn*, *Pp*: *Pn*)	Calculated
	*TR*	Transfer rate	87.4%	Based on the open scientific literatures to calculate
			22.1%	
			2.8%	
	*Cons*	The consumption of tea	*Invgauss* (3.772, 0.721)^a^	China National Nutrient and Health Survey
			*Invgauss* (4.474, 1.718)^b^	
			*Invgauss* (11.532, 10.870)^c^	
	*BW*	Body weight for tea consumers	*Normal* (55.1, 15.5)	China National Nutrient and Health Survey

For *Conc*_*Qp*_ and *Conc*_*Qn*_ mark with an asterisk (*), RPFs were calculated based on CED at 20% AChE inhibition in rat brain, IC for OPs is methamidophos

For *Conc*_*Qp*_ and *Conc*_*Qn*_, RPFs were calculated based on BMDat 10% AChE inhibition in rat brain, IC for OPs is methamidophos

The 87.4, 22.1 and 2.8% were TRs of soluble OPs, moderately soluble OPs and low soluble OPs, respectively

^a^Non-fermented tea

^b^Semi-fermented tea

^c^Fermented tea

## Results

### Residue and food consumption data

Table 2 summarises the results of the samples analysed, including the positive rates and concentrations of detected samples in tea for all types of OPs. In total, 1687 tea samples were analysed for 25 OP pesticides in the National Monitoring Program. There were 169 positive tea samples tested for 20 OPs, including highly toxic pesticides, e.g. methamidophos, parathion-methyl, parathion, monocrotophos, triazophos; moderately toxic pesticides, e.g. chlorpyrifos, omethoate, dimethoate; and low toxicity pesticides, e.g. acephate and malathion. The total positive rate was 10.02%. Chlorpyrifos (6.75%), triazophos (2.59%) and disulfoton (0.74%) were detected most among the 20 OPs in the tea samples above the LOD. The highest concentration values were found for chlorpyrifos (1.900 mg/kg), dimethoate (0.980 mg/kg) and omethoate (0.930 mg/kg). As the monitored tea samples were found to have less pyridaphethione, diazinon, trichlorfon, quinalphos and phoxim, and the positive rate was 0, this study included 20 detected OP residues in its cumulative exposure assessment.

**Table 2:** Concentrations of OPs in tea from the 2013–2014 National Monitoring Program

OPs	Samples analysed	Detected samples	Positive rate (%)	Concentrations of detected samples in tea (mg/kg)					
				Mean	P_50_	P_95_	P_97.5_	P_99.9_	Max
Chlorpyrifos	1660	112	6.75	0.138	0.060	0.594	0.788	1.900	1.900
Triazophos	1619	42	2.59	0.152	0.125	0.427	0.801	0.830	0.830
Disulfoton	1623	12	0.74	0.265	0.295	0.706	0.706	0.706	0.706
Dimethoate	1642	11	0.67	0.261	0.220	0.980	0.980	0.980	0.980
Monocrotophos	1634	10	0.61	0.201	0.095	0.909	0.909	0.909	0.909
Parathion-methyl	1646	10	0.61	0.052	0.041	0.162	0.162	0.162	0.162
Ethion	1638	9	0.55	0.066	0.020	0.240	0.240	0.240	0.240
Tolclofos-methyl	386	2	0.52	0.080	0.080	0.080	0.080	0.080	0.080
Acephate	1635	8	0.49	0.033	0.031	0.077	0.077	0.077	0.077
Methamidophos	1640	8	0.49	0.057	0.033	0.120	0.120	0.120	0.120
Dichlorvos	1638	7	0.43	0.055	0.014	0.130	0.130	0.130	0.130
Omethoate	1628	6	0.37	0.068	0.070	0.930	0.930	0.930	0.930
Fenitrothion	1638	6	0.37	0.033	0.016	0.100	0.100	0.100	0.100
Parathion	1642	6	0.37	0.024	0.008	0.073	0.073	0.073	0.073
Phorate	1649	6	0.36	0.006	0.003	0.017	0.017	0.017	0.017
Chlorpyrifos-methyl	1598	5	0.31	0.020	0.005	0.080	0.080	0.080	0.080
Phosalone	1616	5	0.31	0.047	0.025	0.152	0.152	0.152	0.152
Methidathion	1629	5	0.31	0.076	0.044	0.170	0.170	0.170	0.170
Phosmet	1575	3	0.19	0.013	0.013	0.013	0.013	0.013	0.013
Malathion	1645	3	0.18	0.012	0.012	0.012	0.012	0.012	0.012
Pyridaphethione	40	0	0	–	–	–	–	–	–
Diazinon	6	0	0	–	–	–	–	–	–
Trichlorfon	3	0	0	–	–	–	–	–	–
Quinalphos	3	0	0	–	–	–	–	–	–
Phoxim	3	0	0	–	–	–	–	–	–

As the same tea sample may simultaneously contain more than one type of OP, Table 3 shows the tea samples that were positive for the detection of a number of OPs. Among 169 samples that tested positive for OPs, a total of 129 (7.65%) tea samples contained only one OP, 27 (1.60%) tea samples contained two pesticides and 13 (0.77%) contained three or more OPs.

**Table 3:** Summary of residue data of OP pesticides detected on tea samples analysed in China Monitoring Program

Number of detected pesticides in samples	Non-fermented tea	Semi-fermented tea	Fermented tea
	Number of detected samples	Number of detected samples	Number of detected samples
0	1031	230	257
1	84	33	12
2	21	1	5
3	4	1	0
4	2	0	0
≥ 5	5	1	0
Total of detected samples	116	36	17
Total of number of samples	1147	266	274

Table 4 shows national consumption for three types of dry tea leaves according to the China National Nutrient and Health Survey (2002). Fermented tea showed the highest consumption level out of the three types of tea (9.83 g/d). Non-fermented tea and semi-fermented tea showed an average of 3.92 (g/d) and 4.42 (g/d), respectively. Fermented tea was consumed by the highest percentage of tea consumers (66%); non-fermented tea and semi-fermented tea consumers accounted for 31.2 and 2.8%, respectively.

**Table 4:** Detailed consumption data of tea consumers in China

Tea category	Consumption of dry tea leaves (g/d)					
	Mean	P50	P90	P95	P97.5	Max
Non-fermented green tea	3.92	1.70	11.09	16.70	25.00	41.70
Partly fermented oolong tea	4.42	3.40	9.98	10.00	10.00	10.00
Fermented black tea	9.83	10.00	19.80	25.00	48.80	61.90

*P* percentile, *Max* maximum

### Dietary exposure assessment

Table 5 shows the results of the distribution of exposure to OP residues from tea infusions for tea consumers in China. RPFs were calculated by BMD at 10% AChE inhibition in the female rat brain, and CED at 20% AChE inhibition in the female rat brain; the IC for OPs is methamidophos. Both types of critical effect doses were calculated, and the exposure distributions were extremely right-skewed, with estimates at P99.9, approximately more than two times higher than P99. This is because both food consumption and OP residue monitoring distributions were right-skewed, and the results of the exposure distribution were also right-skewed [49].

**Table 5:** Percentiles of distribution for dietary intake of Ops residues from tea infusion for tea consumers only in China

Percentiles of exposure	OPs^a^ (μg/kg bw/d)	OPs^b^ (μg/kg bw/d)
Tea consumer		
Mean	0.02 (0.02–0.02)^c^	0.08 (0.08–0.09)^c^
P50	0.01 (0.01–0.01)	0.05 (0.05–0.05)
P90	0.04 (0.04–0.06)	0.17 (0.17–0.25)
P95	0.06 (0.05–0.06)	0.25 (0.24–0.25)
P97.5	0.08 (0.07–0.08)	0.33 (0.32–0.35)
P99	0.11 (0.10–0.12)	0.46 (0.44–0.50)
P99.9	0.25 (0.20–0.30)	1.08 (0.85–1.23)

^a^RPFs calculated by BMD_10_, IC for OPs is methamidophos

^b^RPFs calculated by CED_20_, IC for OPs is methamidophos

^c^Numbers with brackets are the lower (2.5%) and upper (97.5%) bounds of the 95% confidence interval of the corresponding percentiles of exposure

### Sensitivity analysis

**Fig. 1:**
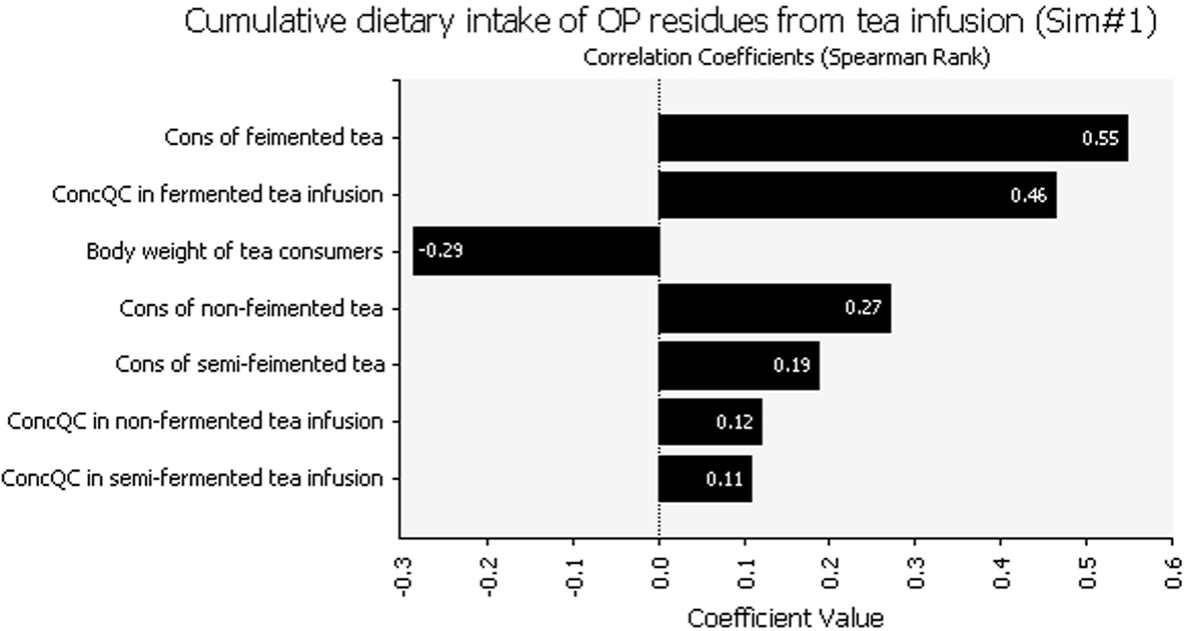
Sensitivity analysis of cumulative dietary exposure to OP residues from tea infusion

A sensitivity analysis was performed using @Risk software by calculating the Spearman rank-order correlation coefficient among the concentrations in the IC equipotent of OP residues in tea infusion, tea consumption and body weight. Figure 1 is the tornado diagram of sensitivity analysis of cumulative dietary exposure to OP residues from tea infusion. The absolute value of the correlation coefficient represents the ability of this variable to influence the cumulative dietary exposure to OP residues from tea infusion (Fig. 1). The results of the sensitivity analyses showed that the consumption of fermented tea was the most influential variable on cumulative dietary exposure to OP residues from tea infusion (correlation coefficient equal to 0.55). The second most influential variable was the concentration in methamidophos equivalents of OP residues in fermented tea infusions (correlation coefficient equal to 0.46).

## Discussion

### Dietary exposure assessment

**Fig. 2:**
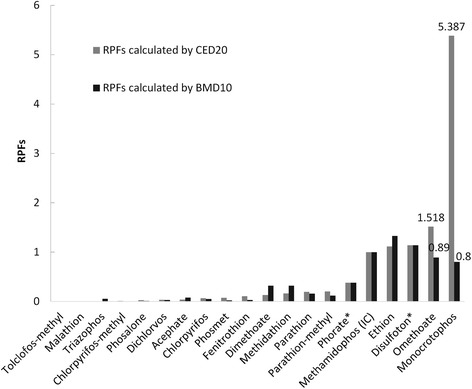
Two types of RPFs calculated by BMD_10_ and CED_20_ inhibition in the female rat brain after 21 days exposure to OPs

Table 5 demonstrates that RPFs were calculated by CED_20_ in the female rat brain to estimate dietary exposure to OP residues from tea infusion was higher than the exposure results that were calculated by RPFs based on BMD_10_ in the female rat brain. The RPF approach assumes that the chemicals under consideration (1) act by a common mode of action, (2) differ only in potency and (3) do not interact [37]. If the log (dose)-response curves are parallel, the RPFs should be constant over the range of effects; RPFs were calculated by BMD_10,_ and CED_20_ should be the same in theory. Compared to the two types of RPFs calculated by BMD_10_ and CED_20_ inhibition in the female rat brain after 21 days exposure to OPs, with methamidophos as the IC for OPs, the RPFs of all the OPs were almost identical, except for monocrotophos and omethoate (Fig. 2). Therefore, we chose the RPFs that were calculated by CED_20_ in the female rat brain to estimate the dietary exposure to OP residues from tea infusion in order to not underestimate the risk.

### Sensitivity analysis

In a sensitivity analysis, the absolute value of the correlation coefficient of the consumption of fermented tea was highest of all. The higher absolute value of the correlation coefficient shows that the factor has the greater influence to the dietary exposure to OP residues from tea infusion.

But in our study, for tea consumers (especially extreme tea consumers), it may be hard to change one’s habits with regard to consumption volume and types of tea consumed. Therefore, strengthening the management of OP residues in fermented tea would be effective in reducing OP exposure levels for tea consumers. These results may provide guidance for intervention measures for risk management.

### Dietary risk assessment

In exposure assessments of OP residues in tea infusion, according to the regulatory threshold, the risk is at the upper tail of the exposure distribution (usually at P99.9) that is used by the US EPA [50]. The cumulative dietary exposures to OP residues in tea infusion were compared with the ARfD of methamidophos, 10 μg/kg bw/d. In this study, at P99.9, the cumulative dietary exposure to OP residues in tea infusion in methamidophos equivalents for Chinese tea consumers was 1.08 μg/kg bw/d, which accounted for 10.8% of the ARfD. In consideration of the most of the tea samples were undetectable, a worst case of the acute dietary intake of OPs residues from tea infusion was also calculated. Using of a deterministic assessment approach, tea samples with concentrations below the LOD were assumed to be LOD; the P99.9 of acute cumulative dietary intake of OPs residues from tea infusion were 5.85 μg/kg bw/d. This is the most conservative estimation of acute dietary cumulative intake of OP residues from tea infusion, which accounted for 58.5% of the ARfD.

It should be noted, however, that for tea consumers, the health risk of dietary exposure to OP residues should account for tea consumption as well as other dietary contributors (e.g. vegetables, fruits). A cumulative risk assessment study from Sun Jin-fang, which assessed dietary exposure to OP residues in China, also used the RPF approach; methamidophos was the IC, non-detected values were considered as LOD and the estimated means of cumulative dietary exposure (a total of 51 types of food including vegetables, fruits, staple foods) to OP residues for the general population and adults above 18 years old were 0.681 and 0.592 μg/kg bw/d, accounting for 6.81 and 5.92% of the ARfD [35]. In Sun’s study, the vegetables were the largest contributor to OP residue intake in the general population; the tea contributed less than the vegetables for the general population [35]. This is mainly due to their low consumption in frequency and volume of tea among the general population.

According to dietary exposure to OP residues in Sun’s study, considering OP residues from vegetables, fruits and other foods, it can be concluded that the level of acute dietary exposure to OP residues in China for both general tea consumers and high tea consumers is safe. However, evidence suggests an association between low level and chronic exposure of OP pesticides and disorder of psychological and neurological development [51–53]. Therefore, it is necessary to strengthen the regulation of the OP pesticides used on tea in China to reduce the risk of chronic dietary exposure to OPs from tea infusion.

In addition, some uncertainties may have influenced the exposures estimated in the present study. First, we only focused on types of tea for which we had both concentration and consumption data; scented tea, brick tea and tea bags were not included, and this may have underestimated OP exposures for some tea consumers. Second, the tea consumption data used in this study were from the China National Nutrition and Health Survey of 2002, and tea consumption patterns and volume may have changed during the 15-year interval since the study. Meanwhile, there were 20 types of OPs detected in the tea samples in the Monitoring Program. Of these 20 types of pesticides, 7 have been banned for use in tea crops in China since 2005 due to their high toxicity. However, some banned pesticides may still be used in China. Therefore, it is important to implement regular monitoring of tea for pesticide residues and to pay more attention to the management of OP pesticides which are prohibited on tea in China.

## Conclusions

This study provides estimates of cumulative dietary exposures to OPs from tea infusions using tea consumption data from the China National Nutrient and Health Survey (2002) and data on OP residue concentrations from the China National Monitoring Program (2013–2014). @Risk software was used to conduct the cumulative dietary exposure assessment in a probabilistic way. For tea consumers, the mean and P99.9 of cumulative dietary exposure to OP residues from tea infusions equalled 0.08 and 1.08 μg/kg bw/d. When compared with the ARfD, 10 μg/kg bw/d for methamidophos, this accounts for 0.8 and 10.8% of the ARfD. Even when considering OP residues from vegetables, fruits and other foods, there are no health concerns based on acute dietary exposure to OP residues from tea infusion. However, it is necessary to strengthen the regulation of the OP pesticides used on tea in China to reduce the risk of chronic dietary exposure to OPs from tea infusion.

## Additional file

Additional file 1: **Table S1.** BMD at 10% AChE inhibition in female rat brain of OPs found in tea samples from the China monitoring programs. Table S2. CED at 20% AChE inhibition in female rat brain of OPs found in tea samples from the China monitoring programs. Table S3. TRs of OP residues to tea infusion. Table S4. Water solubility and octanol-water partition coefficient of OPs. (DOCX 28 kb)

## Abbreviations

ARfD: Acute reference dose
BMD: Benchmark dose
BW: Body weight
CED: Critical effect dose
EPA: Environmental Protection Agency
GC-ECD: Gas chromatograph-electron-capture detector
GC-FID: Gas chromatograph-flame ionisation detector
GC-FPD: Gas chromatograph-flame photometric detection
GC-MS/MS: Gas chromatography–mass spectrometry
GC-NPD: Gas chromatograph-nitrogen-phosphorus detector
IC: Index compound
JMPR: Joint Meeting of Pesticide Residues
LOD: The limit of detection
NOAEL: No observed adverse effect levels
OP: Organophosphorus
RPF: Relative potency factor
TR: Transfer rate
UHPLC-MS/MS: Ultrahigh-performance liquid chromatography-tandem mass spectrometry
