# Supplementary material for: Estimated assessment of cumulative dietary exposure to organophosphorus residues from tea infusion in China
Source: Environ Health Prev Med. 2018 Feb 14;23:7. doi: 10.1186/s12199-018-0696-1 (PMC5813321; doi:10.1186/s12199-018-0696-1)
Supplement: Supplementary file 1 — Table S1. BMD at 10% AChE inhibition in female rat brain of OPs found in tea samples from the China monitoring programs. Table S2. CED at 20% AChE inhibition in female rat brain of OPs found in tea samples from the China monitoring programs. Table S3. TRs of OP residues to tea infusion. Table S4. Water solubility and octanol-water partition coefficient of OPs. (DOCX 28 kb) [file 12199_2018_696_MOESM1_ESM.docx]

Additional file 1: Table S1

BMD at 10% AChE inhibition in female rat brain of OPs found in tea samples from the China monitoring programmes

| Compound | BMD_10_ or NOAEL  (mg/kg bw) | Effect | Source^c^ | RPF |
| --- | --- | --- | --- | --- |
| Acephate^*^ | 0.99 | Brain/rat | EPA02 | 0.080 |
| Chlorpyrifos^*^ | 1.48 | Brain/rat | EPA02 | 0.050 |
| Chlorpyrifos-methyl^*^ | 16.2 | Brain/rat | EPA02 | 0.005 |
| Dichlorvos^*^ | 2.35 | Brain/rat | EPA02 | 0.030 |
| Dimethoate^*^ | 0.25 | Brain/rat | EPA02 | 0.320 |
| Disulfoton^*^ | 0.07 | Brain/rat | EPA02 | 1.140 |
| Ethion | 0.06^a^ | Brain/dog | JMPR90 | 1.330 |
| Fenitrothion | 2.5 ^a^ | Brain/rat | JMPR00 | 0.030 |
| Malathion^*^ | 313.9 | Brain/rat | EPA02 | 0.0003 |
| Methamidophos^*^ (IC) | 0.08 | Brain/rat | EPA02 | 1.000 |
| Methidathion^*^ | 0.25 | Brain/rat | EPA02 | 0.320 |
| Monocrotophos | 0.1 | Brain/rat | NL03 | 0.800 |
| Omethoate^*^ | 0.09 | Brain/rat | EPA02 | 0.890 |
| Parathion^*^ | 0.5 | Brain/rat | EPA99 | 0.160 |
| Parathion-methyl^*^ | 0.67 | Brain/rat | EPA02 | 0.120 |
| Phorate^*^ | 0.21 | Brain/rat | EPA02 | 0.380 |
| Phosalone^*^ | 6.93 | Brain/rat | EPA02 | 0.010 |
| Phosmet^*^ | 3.56 | Brain/rat | EPA02 | 0.020 |
| Tolclofos-methyl | 79 ^a^ | Brain/rat | JMPR94 | 0.001 |
| Triazophos | 1.5 ^a^ | RBC/rat | JMPR02 | 0.053 |

Note: BMD_10_=benchmark dose at 10% AChE inhibition in rat brain.

^a^ NOAEL=no-observed adverse effect levels.

For OPs mark with an asterisk (*) , were obtained from EPA(1999,2002). For the remaining OPs, were obtained from monographs of JMPR (1990, 1994, 2000, 2002) and Netherlands (2003).

Additional file 1: Table S2

CED at 20% AChE inhibition in female rat brain of OPs found in tea samples from the China monitoring programmes.

| Compound | CED_20_ | Effect | Source^c^ | RPF |
| --- | --- | --- | --- | --- |
| Acephate | 4.410 | Brain/rat | EPA01 | 0.038 |
| Chlorpyrifos | 2.580 | Brain/rat | EPA01 | 0.065 |
| Chlorpyrifos-methyl | 16.600 | Brain/rat | EPA01 | 0.010 |
| Dichlorvos | 4.650 | Brain/rat | EPA01 | 0.036 |
| Dimethoate | 1.260 | Brain/rat | EPA01 | 0.133 |
| Malathion | 203.000 | Brain/rat | EPA01 | 0.001 |
| Methamidophos(IC) | 0.167 | Brain/rat | EPA01 | 1.000 |
| Methidathion | 1.030 | Brain/rat | EPA01 | 0.162 |
| Parathion-methyl | 0.820 | Brain/rat | EPA01 | 0.204 |
| Phosalone | 5.950 | Brain/rat | EPA01 | 0.028 |
| Phosmet | 2.280 | Brain/rat | EPA01 | 0.073 |
| Monocrotophos | 0.031 | Brain/rat | (JMPR, 2007) | 5.387 |
| Omethoate | 0.110 | Brain/rat | (JMPR, 2007) | 1.518 |
| Parathion | 0.860 | Brain/rat | (JMPR, 2007) | 0.194 |
| Fenitrothion | 1.540 | Brain/rat | (JMPR, 2007) | 0.108 |
| Tolclofos-methyl | 1494.000 | Brain/rat | (JMPR, 2007) | 0.000 |
| Triazophos | 28.000 | Brain/rat | (JMPR, 2007) | 0.006 |
| Ethion | 0.150 | Brain/rat | (JMPR, 2007) | 1.113 |
| Disulfoton^*^ | ---- | Brain/rat | EPA02 | 1.140 |
| Phorate^*^ | ---- | Brain/rat | EPA02 | 0.380 |

Note: CED_20_= at 20% AChE inhibition in female rat brain.

For OPs mark with an asterisk (*) , no information about CED_20_, RPFs were calculated from BMD_10._

Additional file 1: Table S3

*TRs* of OP residues to tea infusion

| OPs | Tea | Detection | OP in tea leaves before leaching (mg/kg^-1^) | OP in tea infusion  (mg/L) | TR (%) | Reference |
| --- | --- | --- | --- | --- | --- | --- |
| Chlorpyrifos | Green tea | GC-NPD | 50 | 1.57±0.02 | 3.14 | Jaggi et al.(2001) |
| Ethion | Green tea | GC-NPD | 50 | 0.40±0.04 | 0.80 | Jaggi et al.(2001) |
| Malathion | Green tea | GC-NPD | 50 | 6.07±0.40 | 12.14 | Jaggi et al.(2001) |
| Parathion-methyl | Green tea | GC-NPD | 50 | 4.98±0.55 | 9.96 | Jaggi et al.(2001) |
| Dimethoate | Green tea | GC-NPD | 50 | 12.92±1.12 | 25.80 | Jaggi et al.(2001) |
| Monocrotophos | Green tea | GC-NPD | 50 | 9.89±1.08 | 19.78 | Jaggi et al.(2001) |
| Phosphamidon | Green tea | GC-NPD | 50 | 16.65±0.38 | 33.3 | Jaggi et al.(2001) |
| Quinalphos | Green tea | GC-NPD | 50 | 4.02±0.18 | 8.04 | Jaggi et al.(2001) |
| Endosulfan | Green tea | GC-NPD | 50 | 0.9±0.0 | 1.80 | Jaggi et al.(2001) |
| Malathion | peppermint leaves | GC-FID | 40 | 24.90 | 62.25 | Ozbey et al. (2007) |
| Fenitrothion | peppermint leaves | GC-FID | 40 | 15.10 | 37.75 | Ozbey et al. (2007) |
| Dimethoate | peppermint leaves | GC-FID | 40 | 36.50 | 91.25 | Ozbey et al. (2007) |
| Chlorpyrifos | peppermint leaves | GC-FID | 40 | 4.58 | 11.45 | Ozbey et al. (2007) |
| Pirimiphos-ethyl | peppermint leaves | GC-FID | 40 | 5.07 | 12.68 | Ozbey et al. (2007) |
| Chlorpyrifos | Black tea | GC-NPD | 14.3 | 1.3 | 9.12 | Manikandan et al.(2009) |
| Ethion | Black tea | GC-NPD | 61.9 | 1.3 | 2.50 | Manikandan et al.(2009) |
| Quinalphos | Black tea | GC-NPD | 15.0 | 1.38 | 9.20 | Manikandan et al.(2009) |
| Chlorpyrifos | Green tea | GC-MS/MS | 218-1935 | ----- | 0.8 | Chen et al. (2014) |
|  | Oolong tea | GC-MS/MS | 37-849 | ----- | 0.7 | Chen et al. (2014) |
| Triazophos | Green tea | GC-MS/MS | 19-906 | ----- | 29.6 | Chen et al. (2014) |
|  | Oolong tea | GC-MS/MS | 15-838 | ----- | 27.1 | Chen et al. (2014) |
| Isocarbophos | Green tea | GC-MS/MS | 17-886 | ----- | 31.4 | Chen et al. (2014) |
|  | Oolong tea | GC-MS/MS | 36-1705 | ----- | 32.2 | Chen et al. (2014) |
| Fenitrothion | Green tea | GC-ECD | 7.269 ± 1.478 | 1.066±0.041 | 14.67 | Cho et al. (2014) |
| Monocrotophos | Tea | UHPLC-MS/MS | ----- | ----- | 87.4 | Wang et al. (2014) |
| Methidathion | Tea | UHPLC-MS/MS | ----- | ----- | 65.4 | Wang et al. (2014) |
| Acephate | Tea | GC-FPD | 18.67-43.02 | ----- | 80.5-88.3 | Pan et al. (2015) |
| Methamidophos | Tea | GC-FPD | 1.40-3.45 | ----- | 89.1-90.1 | Pan et al. (2015) |

Additional file 1: Table S4

Water solubility and octanol-water partition coefficient of OPs

| Compound | Water solubility （mg/L） | *logK_ow_* | Source |
| --- | --- | --- | --- |
| Acephate | 790000 | -0.9 | IPSC ICSC 0748 |
| Methamidophos | 200000 | -0.66 | IPSC ICSC 0176 |
| Dimethoate | 23300 | 0.7 | IPSC ICSC 0741 |
| Omethoate | readily soluble in water | ---- | WHO(1971 ) |
| Monocrotophos | 10000 | 0.2 | IPSC ICSC 0181 |
| Dichlorvos | 10000 | 1.47 | IPSC ICSC 0690 |
| Methidathion | 187 | 2.2 | IPSC ICSC 1659 |
| Malathion | 145 | 2.89 | IPSC ICSC 0172 |
| Parathion-methyl | 55-60 | 2.65 | The Pesticide Manual(1987) |
| Diazinon | 60 | 3.11 | IPSC ICSC 0137 |
| Phorate | 50 | 3.9 | IPSC ICSC 1060 |
| Triazophos | 35 | 3.34 | WHO(1983) |
| Phosmet | 30 | 2.83 | IPSC ICSC 0543 |
| Disulfoton | 25 | 4.02 | IPSC ICSC 1408 |
| Fenitrothion | 21 | 3.27 | IPSC ICSC 0622 |
| Parathion | 20 | 3.8 | IPCS ICSC0006 |
| Chlorpyrifos-methyl | 4 | ---- | WHO(1975) |
| Phosalone | 3 | 4.3 | IPCS ICSC 0797 |
| Chlorpyrifos | 2 | 4.21 | IPCS ICSC 0851 |
| Ethion | 1 | 5.073 | IPCS ICSC 0888 |
| Tolclofos-methyl | 0.3-0.4 | 4.56 | WHO(1994) |
